# News and Miscellany

**Published:** 1904-08

**Authors:** 


### News and Miscellany.

Dr. John Punton, superintendent of the Punton Sanitarium or Home for Nervous Invalids at Kansas City, is adding a large addition to the sanitarium building in response to an increased demand for accommodation by patients. There is also being built a large extension to the verandas, which will be used by the guests as places of recreation. The management of the Sanitarium highly appreciates the support received from the medical profession and has great confidence in the continued success of the institution.

---

Tulane Medical Department.—The Tulane Medical Department has made several changes in the teaching staff. Through the resignation of Dr. L. F. Reynaud the chair of Materia Medica and Therapeutics was made vacant. The Faculty elected in his stead Dr. J. T. Halsey of the McGill University, Montreal, Can. In addition to this the following have been elected associate professors: Dr. Isadore Dyer to the chair on Diseases of the Skin; Dr. E. D. Fenner to the chair on Diseases of Children; Dr. J. B. Elliott, Jr., to the chair on Clinical Medicine. Dr. P. E. Archinard, in addition to his branch of bacteriology, has been made lecturer, and Gordon King has been made lecturer and clinical instructor on diseases of the ear, nose, and throat; and Dr. J. B. Guthrie has been made lecturer on materia medica and theraputics.

---

The Fumigation of Public Buildings.—The suggestion offered by Health Commissioner Darlington of New York of fumigating churches, theaters, and all buildings used for public gatherings immediately after their use is an excellent one. The majority of colds are contracted not so much from the much dreaded draughts of cold air as from the intimate contact of some one suffering from a coryza. Theaters and churches are as a rule notorious for their inadequate system of ventilation. To enter one of them from the pure outside air about the middle of the season is to be struck at once with the foulness of the atmosphere. That this foul air is the cause of the majority of colds, influenzas, and other air-borne diseases there is no doubt. In the interest of public health ordinances should be passed compelling theater managers to at least thoroughly air their houses after each performance, and at stated intervals, say once a week, to thoroughly disinfect them with formaldehyde.—*Medical Age.*

---

Will Repair Your Electrical Machines.—Static and all electrical medical apparatus put in running order. I am also agent for electrical and X-Ray apparatus. Oliver Brush, 710 Colorado Street, Austin, Texas.

---

Dr. R. H. L. Bibb, so well known and deservedly popular in Texas, his old home, and for many years chief surgeon of the Mexican National Railroad, has been appointed chief surgeon of the combined hospital services of the Mexican National, the International, and the Inter-Oceanic Railroads. These companies have been merged under the "National's" management, and operates 3600 miles of road. Dr. Bibb is in charge of the hospital service of the entire system, including some thirty odd divisions and local surgeons and six general hospitals. The system will, under Dr. Bibb's supervision, at once erect additional hospitals. His headquarters will still be, as at present and in the past, at Saltillo. The medical profession of Texas will be glad to hear of Dr. Bibb's deserved promotion, and the JOURNAL, on behalf of his hosts of friends in Texas, extends its congratulations to the management on securing so efficient a chief, and so distinguished and popular a gentleman for the responsible position.

---

GOOD LOCATION AVAILABLE.—For sale, a large fine residence, barn and stables, separate office near by. Seven acres of fine land. Price, $3000; half cash, balance to suit. Practice averages $2500 to $3000. Collections 98 per cent. Practice goes with property. Pretty village in a beautiful and rich farming section peopled with German and Bohemian settlers; prompt pay. Nearest competition ten miles. Dr. Zvesper, Route 3, La Grange, Texas.

---

MARRIED in Austin, Texas, July 31st (ult.), Mr. M. C. Cornwell to Gertrude, youngest daughter of Dr. F. E. Daniel. No cards.

---

NEW ORLEANS POLYCLINIC—Eighteenth annual session opens November 7, 1904, and closes May 20, 1905. Physicians will find the Polyclinic an excellent means for posting themselves upon modern progress in all branches of medicine and surgery. The specialties are fully taught, including laboratory and cadaveric work. For further information, address New Orleans Polyclinic, postoffice box 797, New Orleans, La.

---

THE TEXAS SANITARIUM AT LLANO will be opened for patients August 15th inst. Llano furnishes the ideal climate for consumptives, and the Sanitarium, a splendid building just finished, is situated in a beautiful natural park of sixty acres. It is 1150 feet above sea level and 150 feet above the level of the beautiful Llano river, which it overlooks. There are tents scattered throughout the park for the more advanced cases. Everything is fully modern and high class. Dr. J. T. Barnard is the Resident Physician; Dr. M. M. Smith, Austin, is Secretary and Treasurer, to whom application should be made for terms and all particulars.

---

THE LUNATIC ASYLUM at Austin won, by competitive bid, the State's contract for many thousand pairs of hose and half hose for the numerous State institutions. Superintendent Worsham has combined business with treatment and put many of his patients to work on knitting machines, and they turn out first-class work. The employment is curative as well as lucrative. The institution is largely self-supporting, and is a credit to the State and to Dr. Worsham and his able assistants who have made it so.

---

ALL DOWN.—Mrs. Newlywed—Doctor, that bottle of medicine you left for baby is all gone.

Doctor—Impossible! I told you to give him a teaspoonful once an hour.

Mrs. Newlywed—Yes; but John and I and mother and the nurse have each to take a teaspoonful, too, in order to induce baby to take it.—*Puck*.

---

DR. GEO. R. TABOR, State Health Officer of Texas, has transferred headquarters of the Department of Public Health and Vital Statistics from Austin to Galveston for the summer, closing the office at Austin and taking his Secretary with him. He will have personal charge of the Galveston Quarantine Station, relieving Dr. McClendon, the officer in charge, who has been granted a leave of absence. Dr. Tabor is considerably pulled down in consequence of his arduous labors, and it is hoped that two months on the Gulf coast, out at the cool quarantine station, with good fishing, bathing and boating, will be beneficial to him.

---

DR. J. T. WILSON of Sherman, who has served the State Medical Association so efficiently on the legislative committee, has been appointed by the president of the association as Texas member on the National Legislative Council. Dr. C. A. L. Reed of Cincinnati is chairman.

